# Including dental professionals in the multidisciplinary treatment team of head and neck cancer patients improves long-term oral health status

**DOI:** 10.1007/s00784-021-04276-x

**Published:** 2021-11-18

**Authors:** Kristina Bertl, Philippe Savvidis, Edmund Benjamin Kukla, Steffen Schneider, Konstantin Zauza, Corinna Bruckmann, Andreas Stavropoulos

**Affiliations:** 1 Department of Periodontology, Faculty of Odontology, University of Malmö, Malmö, Sweden; 2 Division of Oral Surgery, University Clinic of Dentistry, Medical University of Vienna, Vienna, Austria; 3 Comprehensive Center Unit, University Clinic of Dentistry, Medical University of Vienna, Vienna, Austria; 4 Department of Cranio-, Maxillofacial and Oral Surgery, Medical University of Vienna, Vienna, Austria; 5 Division of Conservative Dentistry and Periodontology, University Clinic of Dentistry, Medical University of Vienna, Vienna, Austria; 6 Division of Regenerative Dentistry and Periodontology, University Clinics of Dental Medicine (CUMD), University of Geneva, Geneva, Switzerland

**Keywords:** Head and neck neoplasms, Periodontitis, Dental caries, Dental care, Quality of life

## Abstract

**Objective:**

To assess in a cross-sectional study the impact of including dental professionals in the multidisciplinary treatment team of head and neck squamous cell carcinoma (HNSCC) patients on the long-term oral health status.

**Materials and methods:**

Oral health status, dental care behaviours, and oral health–related quality of life were assessed based on a clinical and radiographic examination, interview, and medical records in patients treated for HNSCC ≥ 6 months ago. This patient group (‘cohort 2’) was treated in a multidisciplinary treatment team including dental professionals and compared to a group of HNSCC patients previously treated at the same university, but without dental professionals included in the multidisciplinary treatment team (‘cohort 1’).

**Results:**

Cohort 2 consisted of 34 patients, who had received a dental check-up and if necessary, treatment by dental professionals prior to the initiation of cancer treatment. This cohort showed significantly improved oral hygiene habits and a better periodontal health status compared to cohort 1. However, cohort 2 still presented high demand for treatment due to active carious lesions; only a few, statistically insignificant improvements were detected compared to cohort 1.

**Conclusion:**

Including dental professionals in the multidisciplinary treatment team of HNSCC patients has a positive impact on patient oral health status—primarily in terms of periodontal disease—6 months and longer after finishing cancer therapy.

**Clinical relevance:**

A team-based approach including dental professionals specialised in head and neck cancer improves oral health status.

**Supplementary Information:**

The online version contains supplementary material available at 10.1007/s00784-021-04276-x.

## Introduction

Assessment of dental treatment needs and delivery of treatment after diagnosis and prior to treatment initiation of head and neck squamous cell carcinomas (HNSCC) is considered an integral part in the overall treatment plan [1–8]. Eradication of oral foci prior to cancer treatment should be performed in a timely manner without delaying initiation of cancer treatment [2, 5, 7]. Patients being diagnosed with HNSCC present on average with a high prevalence of dental treatment needs (i.e. 58–97%) primarily regarding periodontal disease and caries [9–15]. Lack of eradication of oral foci, poor oral hygiene, and poor dental and periodontal conditions increase the rate of side effects of HNSCC treatment (e.g. wound failure, development of osteoradionecrosis (ORN)) [14, 16–19] and might even negatively affect the mortality rate [20]. Hence, several reviews and recommendations have been recently published about the timepoint and extent of dental interventions during and after cancer treatment, to minimise the side effects caused by a poor oral health status [2, 3, 5–8]. Inclusion of dental professionals in multidisciplinary treatment teams for HNSCC is considered to provide advantages in terms of secure and fast eradication of oral foci, not only prior to but also during and after cancer treatment [21–23]. This may be facilitated through, for example, a more simple and direct communication with the patient, faster handling of the patient, better information on the intended cancer treatment protocol (e.g. region and intensity of radiation), etc., compared to a situation where the patient is simply referred to or advised to visit his/her regular dentist.

In this context, a previous publication [24] reported on a cohort of HNSCC patients treated in a tertiary hospital, where dental professionals were not included in the multidisciplinary treatment team. Almost half of the patients did not receive a dental check-up in the timeframe between diagnosis and treatment initiation of HNSCC. After cancer treatment, these patients presented with high dental treatment needs, although 69% of the patients had consulted a dentist within the last year. Eighty-eight percent of the patients still required dental treatment, with 75% of them having ≥ 1 tooth with caries and 78% having periodontitis. Five years ago, a group of dental professionals specialised in the treatment of cancer patients was included in the multidisciplinary treatment team of this tertiary hospital. The aim of the present study was to assess oral health status, dental care behaviours, and oral health–related quality of life (OHRQoL) ≥ 6 months after cancer therapy in HNSCC patients, that had the dental professionals already included in the multidisciplinary treatment team (i.e. cohort 2), and compare them to the patient cohort mentioned above (cohort 1), where dental professionals had not been part of the team [24].

## Materials and methods

### Patient recruitment and inclusion criteria

The Comprehensive Center Unit (CCU; University Clinic of Dentistry, Medical University of Vienna, Austria) is a newly established department with dental professionals specialised in the treatment of cancer patients. It receives referrals from the nearby hospitals for the assessment of dental treatment needs prior to cancer treatment, as well as for dental treatment support during and after cancer treatment. Patients who (1) were diagnosed with HNSCC (i.e. in the oral cavity, nasopharynx, oropharynx, hypopharynx, larynx, or any combination thereof); (2) were fully or partially dentate; (3) > 18 years old; (4) had received a dental check-up (and if required, treatment) prior to cancer treatment at this specific department; and (5) had finished cancer treatment ≥ 6 months ago were invited to participate (cohort 2). The present cross-sectional study was approved by the ethics committee of the Medical University of Vienna (EK-Nr: 2052/2017) and reporting complies with the STROBE guidelines (Supplementary Table 1).

### Data collection based on medical records and questionnaire

The following patient and HNSCC characteristics were retrieved from the medical records: (1) age; (2) gender; (3) registered physical address; (4) site of primary tumour; (5) type of cancer treatment (radio-, chemo-, and/or surgical therapy); and (6) time passed since cancer treatment (months). Based on a combination of the medical records and a personal interview, the following information on patient general habits, and dental treatment needs and care behaviours prior to and after cancer treatment was collected: (1) smoking status (never/former/current smoker); (2) alcohol consumption (frequency); (3) socioeconomic status (family status; monthly income); (4) education level (no education/school/high school/university); (5) dental treatment performed prior to and after cancer treatment; (6) time passed since last dental check-up; and (7) oral hygiene habits (frequency of tooth brushing; type of toothbrush; brushing time; use of an interdental cleaning device, tongue cleaner and/or mouthwash; frequency of professional tooth cleaning). OHRQoL was assessed by a standardised questionnaire (Oral Health Impact Profile; OHIP-G 14). Similar to previous studies [25, 26], answers were assessed based on a 5-point Likert scale (i.e. never/seldom/occasionally/often/very often) as well as by a simple count scoring method, where an impact and score of 1 was recorded if either ‘occasionally’, ‘often’, or ‘very often’ was met, i.e. resulting in a maximum sum of 14.

### Data collection based on clinical and radiographic examination

A single examiner (PS) recorded the following clinical parameters at the remaining dentition (including the wisdom teeth): (1) number of teeth; (2) number of teeth with caries; (3) plaque index in % (PI; plaque control recorded according to O-Leary et al. [27], evaluated at 6 sites/tooth); (4) bleeding on probing in % (BoP; evaluated on 6 sites/tooth 30 s after probing); (5) probing pocket depth in mm (PD; evaluated at 6 sites/tooth); and (6) clinical attachment level in mm (CAL; evaluated at 6 sites/tooth). Periodontal disease was judged as follows: ‘absent’; ‘slight’ [≥ 2 interproximal sites with attachment loss ≥ 3 mm and ≥ 2 interproximal sites with PD ≥ 4 mm (not on same tooth) or one site with PD ≥ 5 mm]; ‘moderate’ [≥ 2 interproximal sites with attachment loss ≥ 4 mm (not on same tooth) or ≥ 2 interproximal sites with PD ≥ 5 mm (not on same tooth)]; ‘severe’ [≥ 2 interproximal sites with attachment loss ≥ 6 mm (not on same tooth) and ≥ 1 interproximal site with PD ≥ 5 mm] according to the criteria by Eke et al. [28].

At the timepoint of clinical examination, an orthopantomogram was taken and the following parameters were assessed at each tooth in a radiographic image analysis software: (1) radiographic alveolar bone loss (ABL) expressed as ‘percentage’ [(radiographic attachment loss/root length)*100; radiographic attachment loss was the average of the distance from the cemento-enamel junction (or the restoration margin) to the alveolar bone crest mesially and distally at each tooth, and the root length was measured from the cemento-enamel junction (or the restoration margin) to the root apex]; (2) number of root canal–treated teeth; (3) number of periapical pathologies (radiolucency in connection with the apical part of the root, exceeding at least twice the width of the lateral part of the periodontal ligament) [29, 30]; (4) number of residual roots; (5) number of dental cysts; and (6) number of impacted teeth.

### Statistical analysis

Data were described descriptively and means (standard deviation), median (first and third quartile), and frequency distributions were calculated; the distribution of the continuous variables was tested by the Shapiro–Wilk test. The data recorded from cohort 2 were compared to the previously published data from cohort 1 [24]; any differences between the 2 study cohorts were assessed by the Mann–Whitney *U* test (if not normally distributed) or by the independent *t*-test (if normally distributed) and chi-squared test was applied for comparison of frequency distributions. In cohort 2, additional comparisons with the same statistical methods were performed between patients continuing with dental treatment at the CCU and those continuing with a general dentist. Any correlations between the OHIP scores and the clinical parameters were assessed by Pearson’s correlation coefficient. Statistical analysis was performed with STATA/IC 16.0 for Mac and a *p*-value of ≤ 0.05 was considered statistically significant.

## Results

### Patient and HNSCC characteristics

Altogether, 216 patients, who had been treated at some timepoint at the above-mentioned department and potentially fulfilled the eligibility criteria, were contacted, and asked for participation. Except for a single person, all had their physical address in Austria. Specifically, about 79% had their physical address in Vienna, about 15.4% within 50 km to Vienna, 3.7% within 50 to 100 km to Vienna, and only 1.9% more than 100 km away from Vienna. Ninety-three patients could not be reached, 59 denied participating via telephone, 18 were deceased, and 46 were invited for clinical evaluation. However, 12 of those 46 did either not fulfil all eligibility criteria or denied participating in all parts of the examination; hence, 34 patients (7 females/27 males; mean age: 60.1 ± 12.9 years) could finally be included, constituting cohort 2. At the timepoint of examination, about half of the participants were former smokers and drinking alcohol less than once per month. Furthermore, about two-thirds of the participants were living in a relationship, had finished a basic education, and had a monthly income between 1500 and 3000 €. The only statistically significant difference between the two cohorts was detected for the monthly income, with fewer participants having < 1000 € per month in cohort 2 compared to cohort 1 (Table 1).

**Table 1:** Patient characteristics

		Inclusion of dental professionals in the multidisciplinary treatment team	
		No (cohort 1; *n* = 48)	Yes (cohort 2; *n* = 34)
Age (years)			
	Mean ± S.D	57.9 ± 12.2	60.1 ± 12.9
	Median (Q1; Q3)	58.0 (52.4; 65)	58.5 (54.0; 71.0)
Gender [*n* (%)]			
	Female	14 (29.2)	7 (20.6)
Smoking status [*n* (%)]			
	Never smoker	11 (22.9)	12 (35.3)
	Former smoker	29 (60.4)	16 (47.1)
	Current smoker	8 (16.7)	6 (17.6)
Alcohol consumption [*n* (%)]			
	≥ 4-times/week	5 (10.4)	2 (5.9)
	2–3-times/week	7 (14.6)	4 (11.8)
	2–4-times/month	11 (22.9)	11 (32.3)
	≤ 1/month	25 (52.1)	17 (50.0)
Family status [*n* (%)]			
	Living alone	13 (27.1)	10 (29.4)
	Flat-sharing community	2 (4.2)	0 (0)
	Living in a relationship	32 (66.7)	23 (67.7)
	Single parent	1 (2.1)	1 (2.9)
Education level [*n* (%)]			
	No education	3 (6.3)	1 (2.9)
	School	29 (60.4)	21 (61.8)
	High school	13 (27.1)	5 (14.7)
	University	3 (6.2)	7 (20.6)
Monthly income [*n* (%)]^1^			
	< 1.000 €	19 (39.6)	5 (14.7)
	1.001–1.500 €	11 (22.9)	7 (20.6)
	1.501–2.000 €	11 (22.9)	9 (26.5)
	2.001–3.000 €	4 (8.3)	12 (35.3)
	> 3.000 €	3 (6.3)	1 (2.9)

^1^A statistically significant difference (*p* < 0.05) in the distribution comparing the previous and the present population has been recorded

*n*, number; *Q1*, first quartile; *Q3*, third quartile; *S.D.*, standard deviation

The oropharynx (41.2%), oral cavity (17.7%), and larynx (17.6%) were the most common sites for HNSCC in cohort 2; although cohort 1 included more patients with HNSCC of the oral cavity, the distribution was not significantly different between the cohorts. In most of the cases (79.4%) a multimodal cancer therapy was performed, which was finished 7–54 months prior to participating; most often a combination of radio- and chemotherapy (50%) or radio- and surgical therapy (23.5%) was applied (Table 2).

**Table 2:** Primary tumour site and cancer treatment details

		Inclusion of dental professionals in the multidisciplinary treatment team	
		No (cohort 1; *n* = 48)	Yes (cohort 2; *n* = 34)
Site of primary tumour [*n* (%)]			
	Oral cavity	19 (39.6)	6 (17.7)
	Nasopharynx	7 (14.6)	3 (8.8)
	Oropharynx	9 (18.7)	14 (41.2)
	Hypopharynx	5 (10.4)	3 (8.8)
	Larynx	7 (14.6)	6 (17.6)
	Overlapping multiple sites	1 (2.1)	2 (5.9)
Cancer treatment [*n* (%)]^1^			
	Radiotherapy	1 (2.1)	7 (20.6)
	Chemotherapy	0 (0.0)	0 (0.0)
	Surgical therapy	9 (18.8)	0 (0.0)
	Radio- and chemotherapy	14 (29.1)	17 (50.0)
	Radio- and surgical therapy	9 (18.8)	8 (23.5)
	Chemo- and surgical therapy	2 (4.1)	0 (0.0)
	Radio-, chemo-, and surgical therapy	13 (27.1)	2 (5.9)
Time passed since cancer treatment (months)			
	Mean ± S.D	21.3 ± 13.4	24.6 ± 11.4
	Median (Q1; Q3)	18.5 (11.5; 27)	21.5 (16; 31)

^1^A statistically significant difference (*p* < 0.05) in the distribution comparing the previous and the present population has been recorded

*n*, number; *Q1*, first quartile; *Q3*, third quartile; *S.D.*, standard deviation

### Dental care behaviours prior to cancer treatment and oral hygiene habits

Per inclusion criterium, all individuals included in cohort 2 had received a dental check-up and the necessary treatment prior to cancer treatment. The treatment procedures most often performed were tooth extractions, followed by restorative procedures and professional tooth cleaning. More than half of the patients have received a professional tooth cleaning at least once per year; this is a significantly higher number compared to cohort 1, where 40% never considered a professional tooth cleaning. Interestingly, compared to cohort 1, an almost 4-times higher proportion of patients in cohort 2 were brushing their teeth only once per day (the majority using a manual toothbrush, i.e. 58.8%), yet the average brushing time was longer and the use of any interdental cleaning device more frequent. Specifically, in cohort 2, the average brushing time was almost 1 min longer and only about 23% of the participants did not use any interdental cleaning device, compared to about 60% in cohort 1 (Table 3).

**Table 3:** Assessment of dental care behaviours prior to cancer treatment and oral hygiene habits

		Inclusion of dental professionals in the multidisciplinary treatment team		*p*-value
		No (cohort 1; *n* = 48)	Yes (cohort 2; *n* = 34)	
Dental consultation prior to cancer treatment [*n* (%)]				
	Yes	25 (52.1)	34 (100)	** < 0.001**
Dental treatment performed in the individuals consulting a dentist prior to cancer treatment [*n* (%)]^1,2^				
	Professional tooth cleaning	8 (18.2)	17 (26.2)	-
	Restorative procedure	10 (22.7)	17 (26.2)	
	Root canal filling	3 (6.8)	2 (3.0)	
	Prosthetic procedure	8 (18.2)	4 (6.1)	
	Tooth extraction	15 (34.1)	25 (38.5)	
	No. of patients without treatment needs	5	0	
Frequency of professional tooth cleaning [*n* (%)]				
	Every 3 months	3 (6.3)	0 (0)	**0.008**
	Every 6 months	10 (20.8)	9 (26.5)	
	Once per year	6 (12.5)	9 (26.5)	
	Less than once per year	10 (20.8)	13 (38.2)	
	Never	19 (39.6)	3 (8.8)	
Frequency of tooth brushing [*n* (%)]				
	Once per day	4 (8.3)	11 (32.4)	**0.017**
	Twice per day	43 (89.6)	23 (67.6)	
	Never	1 (2.1)	0 (0)	
Type of toothbrush [*n* (%)]^3^				
	Manual	36 (76.6)	20 (58.8)	0.060
	Electrical	11 (23.4)	11 (32.4)	
	Both	0 (0.0)	3 (8.8)	
Average brushing time (minutes)				
	Mean ± S.D	2.4 ± 1.0	3.3 ± 1.7	**0.006**
	Median (Q1; Q3)	2.0 (2.0; 3.0)	3.0 (2.0; 4.0)	
Interdental cleaning device [*n* (%)]^1,3^				
	Floss	7 (14.9)	13 (38.2)	**0.014**
	Interdental brushes	10 (21.3)	11 (32.4)	0.239
	Toothpick	9 (19.1)	9 (26.5)	0.405
	No interdental cleaning device	28 (59.6)	7 (23.3)	**0.016**
Use of a tongue cleaner [*n* (%)]				
	Yes	1 (2.1)	4 (11.8)	0.071
Use of a mouthwash [*n* (%)]				
	Yes	21 (43.8)	13 (38.2)	0.618

^1^Multiple answers possible

^2^Percentages are related to the total number of different treatment procedures performed

^3^Based on 47 and 34 patients cleaning their teeth, respectively

Bold values indicate statistical significance

*n*, number; *Q1*, first quartile; *Q3*, third quartile; *S.D.*, standard deviation

### Assessment of dental care behaviours after cancer treatment

Except for 3 patients, all patients in cohort 2 followed the recommendation to seek regular dental treatment also after cancer treatment, with half of them visiting a general dentist and the other half continuing at the CCU; however, 2 patients returning at CCU for check-ups did not receive the recommended dental treatment. The treatment procedures most often received were professional tooth cleaning and restorative procedures, and except for 4 patients, all patients had visited a dentist within the last 12 months (Table 4). These data were not in detail assessed in the previous publication; however, almost one-third of the patients in cohort 1 had not visited a dentist within the last year at the timepoint of assessment and only about one-third had received some prosthetic treatment after cancer treatment.

**Table 4:** Assessment of dental care behaviours after cancer treatment in cohort 2

Dental consultation after cancer treatment [*n* (%)]		
	Yes	31 (91.2)
Location of dental care after cancer treatment [*n* (%)]^1^		
	University clinic	14 (45.2)
	University clinic & general dentist	1 (3.2)
	General dentist	16 (51.6)
Dental treatment performed after cancer treatment [*n* (%)]^1,2,3^		
	Professional tooth cleaning	21 (26.9)
	Restorative procedure	15 (19.2)
	Root canal filling	9 (11.6)
	Fixed dental prosthesis	11 (14.1)
	Removable dental prosthesis	9 (11.6)
	Tooth extraction	10 (12.8)
	Implant installation	3 (3.8)
	No. of patients not receiving any treatment	2
Last dental check-up [*n* (%)]^1^		
	1 month ago	16 (51.6)
	6 months ago	8 (25.8)
	12 months ago	3 (9.7)
	More than 12 months ago	4 (12.9)

^1^Based on the 31 patients consulting a dentist after cancer treatment

^2^Multiple answers possible

^3^Percentages are related to the total number of different treatment procedures performed

*n*, number; *S.D.*, standard deviation

### Oral health status at timepoint of examination

The number of remaining teeth between the 2 cohorts was comparable (*p* = 0.713). Patients in cohort 2 compared to cohort 1 had a PI about 20% lower (*p* = 0.003), but the BoP values were significantly worse in cohort 2 compared to cohort 1 (*p* = 0.005). The clinical and radiographic periodontal parameters were all statistically significantly lower in cohort 2 compared to cohort 1. For example, the mean number of teeth with PD ≥ 5 mm was only 1.4 in cohort 2 compared to 3.1 in cohort 1 and the percentage of patients with ≥ 4 teeth with PD ≥ 5 mm in cohort 2 was only 11.8% compared to 37% in cohort 1 (Table 5). The number of teeth with caries appeared slightly improved but lacked statistical significance and in general cohort 2 displayed still a high proportion of patients with at least one active caries lesion (i.e. 67.5%). The additional radiographic parameters, such as number of root canal–treated teeth, are displayed in Supplementary Table 2; no significant difference between the cohorts was detected.

**Table 5:** Oral health status of cohorts 1 and 2

		Inclusion of dental professionals in the multidisciplinary treatment team		*p*-value
		No (cohort 1; *n* = 48)	Yes (cohort 2; *n* = 34)	
No. of teeth				
	Mean ± S.D	18.2 ± 9.1	19.2 ± 7.5	0.713
	Median (Q1; Q3)	20 (9.5; 26.5)	22 (12; 25)	
Oral hygiene indices				
	Plaque [%; mean ± S.D.; median (Q1; Q3)]	65.6 ± 30.0^1^ 69.5 (53.0; 85.8)	47.0 ± 20.5 46.0 (31.3; 60.9)	**0.003**
	BoP [%; mean ± S.D.; median (Q1; Q3)]	16.2 ± 26.1^1^ 5.4 (0.0; 18.2)	17.3 ± 11.8 16.1 (9.7; 21.0)	**0.005**
Caries prevalence				
	% of patients with ≥ 1 teeth with caries	75.0^2^	67.5	0.474
	No. of teeth with caries [mean ± S.D.; median (Q1; Q3)]	5.1 ± 6.3^2^ 3 (0.5; 6)	4.2 ± 5.2 2.5 (0; 6)	0.500
	No. (%) of patients with…			
	…No tooth with caries	11 (25.0)	11 (32.3)	0.806
	…1 to 3 teeth with caries	13 (29.5)	7 (20.6)	
	…4 to 9 teeth with caries	15 (34.1)	12 (35.3)	
	… ≥ 10 teeth with caries	5 (11.4)	4 (11.8)	
Periodontal parameters				
	No. (%) of patients with…			
	…No periodontitis	10 (21.7)	9 (26.5)	**0.021**
	…Slight periodontitis	0 (0.0)	0 (0)	
	…Noderate periodontitis	13 (28.3)	18 (52.9)	
	…Severe periodontitis	23 (50.0)	7 (20.6)	
	PD [mm; mean ± S.D.; median (Q1; Q3)]	2.6 ± 0.7^1^ 2.4 (2.1; 3.0)	2.0 ± 0.5 1.9 (1.7; 2.0)	** < 0.001**
	No. of teeth with PD ≥ 5 mm [mean ± S.D.; median (Q1; Q3)]	3.1 ± 3.7^1^ 1.5 (0; 6)	1.4 ± 2.9 0 (0; 1)	**0.032**
	% of teeth with PD ≥ 5 mm [mean ± S.D.; median (Q1; Q3)]	20.1 ± 26.3^1^ 8.5 (0; 32.3)	7.4 ± 13.8 0 (0; 8)	**0.033**
	No. (%) of patients with…			
	…No tooth with PD ≥ 5 mm	19 (41.3)	20 (58.8)	**0.040**
	…1 to 3 teeth with PD ≥ 5 mm	10 (21.7)	10 (29.4)	
	… ≥ 4 teeth with PD ≥ 5 mm	17 (37.0)	4 (11.8)	
	CAL [mm; mean ± S.D.; median (Q1; Q3)]	3.2 ± 1.4^1^ 2.7 (2.2; 3.5)	2.2 ± 0.6 2.1 (1.8; 2.4)	** < 0.001**
	No. of teeth with CAL ≥ 5 mm [mean ± S.D.; median (Q1; Q3)]	6.0 ± 5.8^1^ 5 (1; 10)	2.9 ± 3.7 1.5 (0; 4)	**0.014**
	% of teeth with CAL ≥ 5 mm [mean ± S.D.; median (Q1; Q3)]	39.7 ± 37.1^1^ 28.6 (3.2; 71.4)	17.1 ± 22.2 8 (0; 28.6)	**0.011**
Radiographic ABL				
	ABL [%; mean ± S.D.; median (Q1; Q3)]	26.8 ± 10.8^2^ 26.1 (19.8; 30.8)	19.6 ± 4.9 19.3 (16.0; 23.1)	**0.001**
	No. of teeth with ABL ≥ 33 < 66% [mean ± S.D.; median (Q1; Q3)]	3.9 ± 4.3^2^ 2 (1; 7))	2.8 ± 2.4 2 (1; 4)	0.651
	No. of teeth with ≥ 66% ABL [mean ± S.D.; median (Q1; Q3)]	0.2 ± 0.7^2^ 0 (0; 0)	0.03 ± 0.2 0 (0; 0)	0.163

^1^Based on the data of 46 patients

^2^Based on the data of 44 patients

Bold values indicate statistical significance

*ABL*, alveolar bone loss; *BoP*, bleeding on probing; *CAL*, clinical attachment level; *PD*, probing pocket depth; *Q1*, first quartile; *Q3*, third quartile; *S.D.*, standard deviation

In cohort 2, patients continuing receiving dental treatment at the CCU presented with better oral hygiene and a lower number of caries lesions compared to those that continued with a general dentist. Specifically, the mean PI values were 39.3 vs. 50.3% (*p* = 0.123) and only 53 vs. 75% of the patients presented with at least 1 tooth with caries (*p* = 0.208), respectively. The total number of teeth with caries appeared lower for the patients continuing at the CCU (i.e. median (Q1; Q3): 1 (0, 4) vs. 4.5 (0.5; 7); *p* = 0.068). No differences in periodontal status were observed between these 2 subgroups of cohort 2. Furthermore, in regard to the 5 patients having no dental treatment after cancer therapy, all presented with the need of restorative treatment due to carious lesions, and 2 of them presented even a very high number of teeth with active caries lesions (i.e. 15 and 18 teeth).

### Oral health–related quality of life

**Fig. 1:**
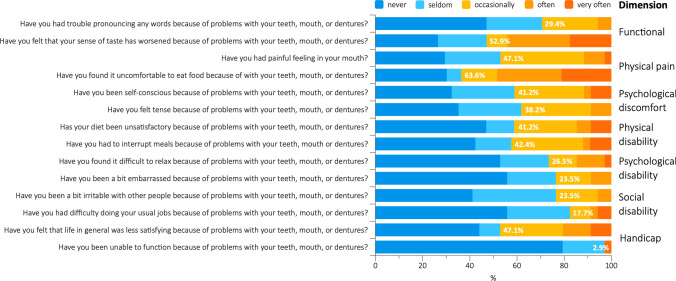
Results of the oral health–related quality of life assessment (OHIP-14). Answers are displayed based on a 5-point Likert scale (i.e. never, seldom, occasionally, often, very often) as well as by a simple count scoring method, where an impact was recorded if either ‘occasionally’, ‘often’, or ‘very often’ was met (i.e. blue/no impact vs. orange/impact; the percentages given in the bars represent the proportion of participants reporting an impact). Two questions (‘Have you had to interrupt meals because of problems with your teeth, mouth or dentures?’ and ‘Have you found it uncomfortable to eat food because of with your teeth, mouth, or dentures?’) have not been answered by one of the participants

The highest impact on OHRQoL in cohort 2 was noted for feeling uncomfortable to eat food (63.6%) and a worsened sense of taste (52.9%). Furthermore, 47.1% of the respondents indicated that life felt in general less satisfying because of problems with teeth, mouth, or dentures, and that they had a painful feeling in the mouth. Least impact was recorded for having difficulties doing the usual jobs (17.7%) and for being totally unable to function (2.9%) (Fig. 1). Overall, the mean impact on OHRQoL was 4.9 ± 3.5 (range: 0–12). No correlation between the impact on OHRQoL and any of the clinical parameters (i.e. PI, BoP, number of teeth with caries, number/percentage of teeth with PD/CAL ≥ 5 mm, number of teeth with ABL ≥ 33%) could be detected (*p* ≥ 0.333). Considering each OHIP question separately, only ‘being totally unable to function because of problems with the teeth, mouth, or dentures’ and BoP showed a statistically significant positive correlation (*r* = 0.587, *p* < 0.001), while the correlation between ‘diet being unsatisfactory because of problems with the teeth, mouth, or dentures’ and the number of teeth with ABL ≥ 33% missed significance (*r* = 0.330, *p* = 0.057).

## Discussion

The present study compared oral health status and dental care behaviours in 2 cohorts of HNSCC patients, who had finished cancer therapy more than 6 months before the examination in the same tertiary hospital. The main difference between the cohorts was that patients in one cohort received treatment by a multidisciplinary team including dental professionals (cohort 2), while patients in the other cohort (cohort 1) were treated for their cancer by a team not including dental professionals and they were only referred to or advised to visit his/her regular (general) dentist [24]. The results showed significant improvements in terms of clinical and radiographic periodontal parameters and better oral hygiene habits in cohort 2, but no significant differences between the cohorts in regard to caries treatment needs.

In particular, almost 60% of the patients in cohort 2 had no teeth with periodontal pockets (i.e. ≥ 5 mm) and only about 12% (i.e. 4 patients) had ≥ 4 teeth (i.e. 7–12 teeth) with a PD ≥ 5 mm; in contrast, almost 40% of the patients in cohort 1 had ≥ 4 teeth with PD ≥ 5 mm. This is an important and relevant improvement, especially when considering that poor periodontal status has been repeatedly described to increase the risk for ORN [7, 15, 31] after tooth extraction. Several studies reported a significant association of plaque score > 40%, PD > 5 mm, and ABL > 60% with ORN development [14, 16, 17], while patients with PD ≥ 6 mm had 5-times more often bone healing problems over a 2-year period after radiotherapy compared to patients with PD < 6 mm (i.e. 19 vs. 4%) [15]. It is believed that the increased risk for ORN in patients with periodontal problems is at least partly due to the excessive bacterial load at periodontally diseased teeth, as periodontal bacteria have been implicated in osteonecrosis in patients on anti-resorptive medication [32]. The improved periodontal conditions observed in cohort 2 should be attributed to the more regular/systematic delivery of periodontal maintenance, including reinforcement in oral hygiene measures. Compared to cohort 1, in which 40% of the patients never received professional tooth cleaning, only a minority (i.e. 9%) of patients in cohort 2 never received a professional tooth cleaning. Furthermore, patients in cohort 2 showed improved oral hygiene attitudes, i.e. they were brushing on average about 1 min longer compared to patients in cohort 1, and the vast majority (i.e. 77%) were using any interdental cleaning device vs. only 40% of the patients in cohort 1. In this context, although there was no difference between the cohorts regarding the number of remaining teeth, the improved periodontal status observed in cohort 2 may be due to a more stringent, including timely, extraction policy of severely affected teeth prior to initiation of cancer treatment, compared to cohort 1. Specifically, only a single patient had a single tooth with an ABL ≥ 66% and the average ABL was significantly lower in cohort 2. However, whether this is really based on a lower extraction rate of periodontally diseased teeth in cohort 1 or on a faster periodontal disease progression after cancer treatment due to less intensive monitoring could not be assessed by the available data.

The patients in cohort 2, similarly to cohort 1 [24] and to what is reported in the literature [9–14], confirmed the high treatment needs in patients being diagnosed with HNSCC (i.e. 58–97%) [9–15], i.e. all patients received some kind of dental treatment with 25 and 17 patients having teeth extracted and receiving restorative procedures, respectively. Only 5 out of 34 patients (i.e. 15%) required only a professional tooth cleaning and oral hygiene instructions prior to initiation of cancer treatment. Interestingly, while the periodontal status appeared clearly improved in cohort 2, this was not the case regarding treatment needs due to carious lesions. Although the average number of teeth with caries was about 1 tooth lower compared to cohort 1 (i.e. 4.2 vs. 5.1 teeth, respectively), this difference was not statistically significant, and the proportion of patients with at least 1 tooth with caries was still quite high (i.e. 67.5%). In this context, the importance of patient compliance in terms of caries development can be evident within a short period of time. For example, in a relatively recent study assessing the importance of oral prophylaxis in HNSCC, patients with low compliance presented 5-times more sites with caries compared to patients with high compliance within only 12 months [33]. Herein, an additional comparison was made within cohort 2 between patients continuing either at CCU or at a general dentist. Those patients continuing at CCU tended to present with better oral hygiene levels and less caries activity, which underlines the importance of close monitoring of HNSCC patients’ oral health within the frames of a multidisciplinary treatment team including dental professionals. However, the relatively small sample size limited this analysis, and this aspect should be further investigated in future studies including a larger population. As mentioned earlier, the idea of including dentists in the multidisciplinary treatment teams is to have professionals with better knowledge of the risk factors of these patients as well as a better understanding on the specific needs and problems of the individual patient, since they can be involved in the whole treatment process. It may also be that HNSCC patients feel more comfortable receiving treatment from the same professional team, compared to from a general dentist and even vice versa. In fact, it has been previously reported that general dental practitioners in > 50% of the cases are ‘not at all’ or only ‘little’ happy about managing cancer patients after treatment [34]. Providing a secure frame for the dental management of HNSCC patients appears necessary also when considering the impact cancer has on the OHRQoL of this group of patients. As demonstrated herein, the majority of the patients in cohort 2 felt uncomfortable to eat food (63.6%) and experienced a worsened sense of taste (52.9%), with a mean overall impact on OHRQoL of approximately 5 about 2 years post-cancer treatment, which in turn is comparable to what was reported previously [26]. Therefore, a team-based approach including dental professionals specialised in head and neck cancer survivors seems appropriate also for post-cancer treatment monitoring, like the recommendations for the period prior to and during cancer treatment [35].

The relatively small sample size should be considered the major limitation of the present study. Almost half of the patients contacted for participation could not be reached and another fourth denied participating, although almost 80% of them had their registered physical address in Vienna and only about 5% had an address > 50 km away from Vienna. This emphasises the need of more effective HNSCC patient communication about the importance and relevance of continuous long-term follow-up. It also shows the need for implementing novel approaches, such as teledentistry, allowing the provision of continuous care for cancer survivors, independent of the possibility of physical visits. Teledentistry has been shown—especially during the COVID-19 pandemic—as helpful and well-accepted tool for many aspects (e.g. motivation for oral hygiene measures, counselling via photographs, advising home care measures) [36]. An interesting aspect regarding oral health status of HNSCC survivors are also any potential differences between younger and older patients, which may—at least partly—depend on their risk profile, i.e. HNSCC in younger patients (< 45 years of age) is more likely associated with human papillomavirus infection, while in older patients alcohol and tobacco consumption remain the main risk factors [37, 38]. Specifically, tobacco consumption is a well-known risk factor also for periodontitis [39] and thereby may affect the oral health status not only prior but also after cancer treatment. However, as only 5 out of 34 patients of cohort 2 were younger than 45 years of age at the timepoint of cancer treatment, no meaningful analysis was possible, and this remains an interesting subject of future studies including a larger patient group. Furthermore, another relevant aspect is the possible impact of ethnicity on the observed results. Vienna is, however, an international city, including a considerable proportion of second and third generation of originally non-Austrians, and thus its population is rather mixed in terms of ethnicity. Thus, we found it difficult to collect reliable information to analyse the possible impact of ethnicity on the observed results.

In conclusion, the present data confirm the general assumption that the inclusion of dental professionals, who are specialised on the treatment of cancer patients, in the multidisciplinary treatment teams of HNSCC patients has a positive impact on oral health status—especially in terms of periodontal disease—6 months and longer after finishing cancer therapy.

## Supplementary Information

Below is the link to the electronic supplementary material.

Supplementary file1 (DOC 83 KB)

Supplementary file2 (DOCX 15 KB)
