# Supplementary material for: Including dental professionals in the multidisciplinary treatment team of head and neck cancer patients improves long-term oral health status
Source: Clin Oral Investig. 2021 Nov 18;26(3):2937–48. doi: 10.1007/s00784-021-04276-x (PMC8600104; doi:10.1007/s00784-021-04276-x)
Supplement: Supplementary file 2 — Supplementary file2 (DOCX 15 KB) [file 784_2021_4276_MOESM2_ESM.docx]

**Supplementary table 2.** Additional radiographic parameters of cohort 1 and 2.

|  | **Inclusion of dental professionals in the multidisciplinary treatment team** | | **p-value** |
| --- | --- | --- | --- |
|  | **No (Cohort 1; n=44)** | **Yes (Cohort 2; n=34)** |  |
| No. of root-canal treated teeth  [mean ± S.D.; median (Q1; Q3)] | 1.7 ± 2.0  1 (0; 3) | 2.2 ± 2.7  2 (0; 2) | 0.361 |
| No. of periapical pathologies  [mean ± S.D.; median (Q1; Q3)] | 0.6 ± 0.9  0 (0; 1) | 0.4 ± 0.8  0 (0; 1) | 0.487 |
| No. of residual roots  [mean ± S.D.; median (Q1; Q3)] | 0.8 ± 3.2  0 (0; 0) | 0.1 ± 0.4  0 (0; 0) | 0.221 |
| No. of dental cysts  [mean ± S.D.; median (Q1; Q3)] | 0.02 ± 0.2  0 (0; 0) | 0.03 ± 0.2  0 (0; 0) | 0.854 |
| No. of impacted teeth  [mean ± S.D.; median (Q1; Q3)] | 0.2 ± 0.5  0 (0; 0) | 0.5 ± 1.0  0 (0; 1) | 0.135 |

*Q1, first quartile; Q3, third quartile; S.D. standard deviation.*
